# Microbial Phagocytic Receptors and Their Potential Involvement in Cytokine Induction in Macrophages

**DOI:** 10.3389/fimmu.2021.662063

**Published:** 2021-04-29

**Authors:** Yan Lin Fu, Rene E. Harrison

**Affiliations:** ^1^ Department of Cell & Systems Biology, University of Toronto, Toronto, ON, Canada; ^2^ Department of Biological Sciences, University of Toronto Scarborough, Toronto, ON, Canada

**Keywords:** macrophage, cytokine, receptor, inflammation, phagocyte

## Abstract

Phagocytosis is an essential process for the uptake of large (>0.5 µm) particulate matter including microbes and dying cells. Specialized cells in the body perform phagocytosis which is enabled by cell surface receptors that recognize and bind target cells. Professional phagocytes play a prominent role in innate immunity and include macrophages, neutrophils and dendritic cells. These cells display a repertoire of phagocytic receptors that engage the target cells directly, or indirectly *via* opsonins, to mediate binding and internalization of the target into a phagosome. Phagosome maturation then proceeds to cause destruction and recycling of the phagosome contents. Key subsequent events include antigen presentation and cytokine production to alert and recruit cells involved in the adaptive immune response. Bridging the innate and adaptive immunity, macrophages secrete a broad selection of inflammatory mediators to orchestrate the type and magnitude of an inflammatory response. This review will focus on cytokines produced by NF-κB signaling which is activated by extracellular ligands and serves a master regulator of the inflammatory response to microbes. Macrophages secrete pro-inflammatory cytokines including TNFα, IL1β, IL6, IL8 and IL12 which together increases vascular permeability and promotes recruitment of other immune cells. The major anti-inflammatory cytokines produced by macrophages include IL10 and TGFβ which act to suppress inflammatory gene expression in macrophages and other immune cells. Typically, macrophage cytokines are synthesized, trafficked intracellularly and released in response to activation of pattern recognition receptors (PRRs) or inflammasomes. Direct evidence linking the event of phagocytosis to cytokine production in macrophages is lacking. This review will focus on cytokine output after engagement of macrophage phagocytic receptors by particulate microbial targets. Microbial receptors include the PRRs: Toll-like receptors (TLRs), scavenger receptors (SRs), C-type lectin and the opsonic receptors. Our current understanding of how macrophage receptor stimulation impacts cytokine production is largely based on work utilizing soluble ligands that are destined for endocytosis. We will instead focus this review on research examining receptor ligation during uptake of particulate microbes and how this complex internalization process may influence inflammatory cytokine production in macrophages.

## Phagocytosis Receptors and Cytokine Induction in Macrophages

**Figure 1:**
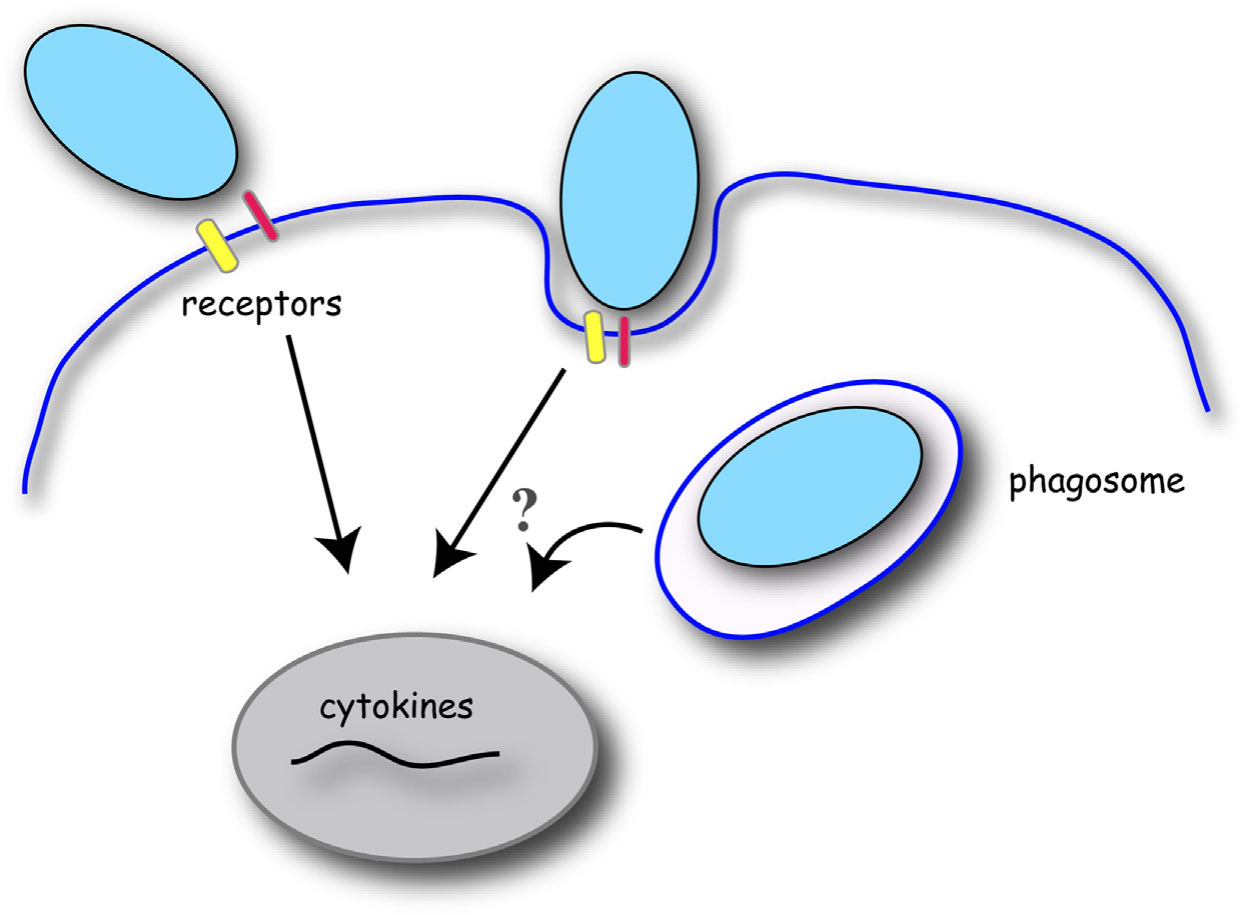
Schematic of phagocytosis and cytokine gene induction in macrophages. Phagocytosis is initiated by the binding of target particles to the macrophage cell surface *via* specific receptors. Receptor signaling initiates localized changes in the plasma membrane and underlying cytoskeleton to engulf and internalize the target particle into a membrane-bound phagosome. Receptor ligation to the target particle induces signal transduction to promote gene expression of pro- or anti-inflammatory cytokines. Conflicting evidence exists for the relevance of particle internalization and phagosome maturation in cytokine production in macrophages.

Phagocytosis is a receptor-mediated process designed to engulf and destroy large target cells, including microbes, from the body. Professional phagocytes possess specialized receptors that can engage with the target directly or *via* intermediary products including opsonins. We will review known phagocytic targets for each receptor and the literature documenting cytokine induction during phagocytosis in macrophages (**Figure 1**). The mechanism by which an inflammatory outcome is signaled is unique to each receptor and we will consider some examples.

### Toll-Like Receptors (TLRs) and CD14

**Figure 2:**
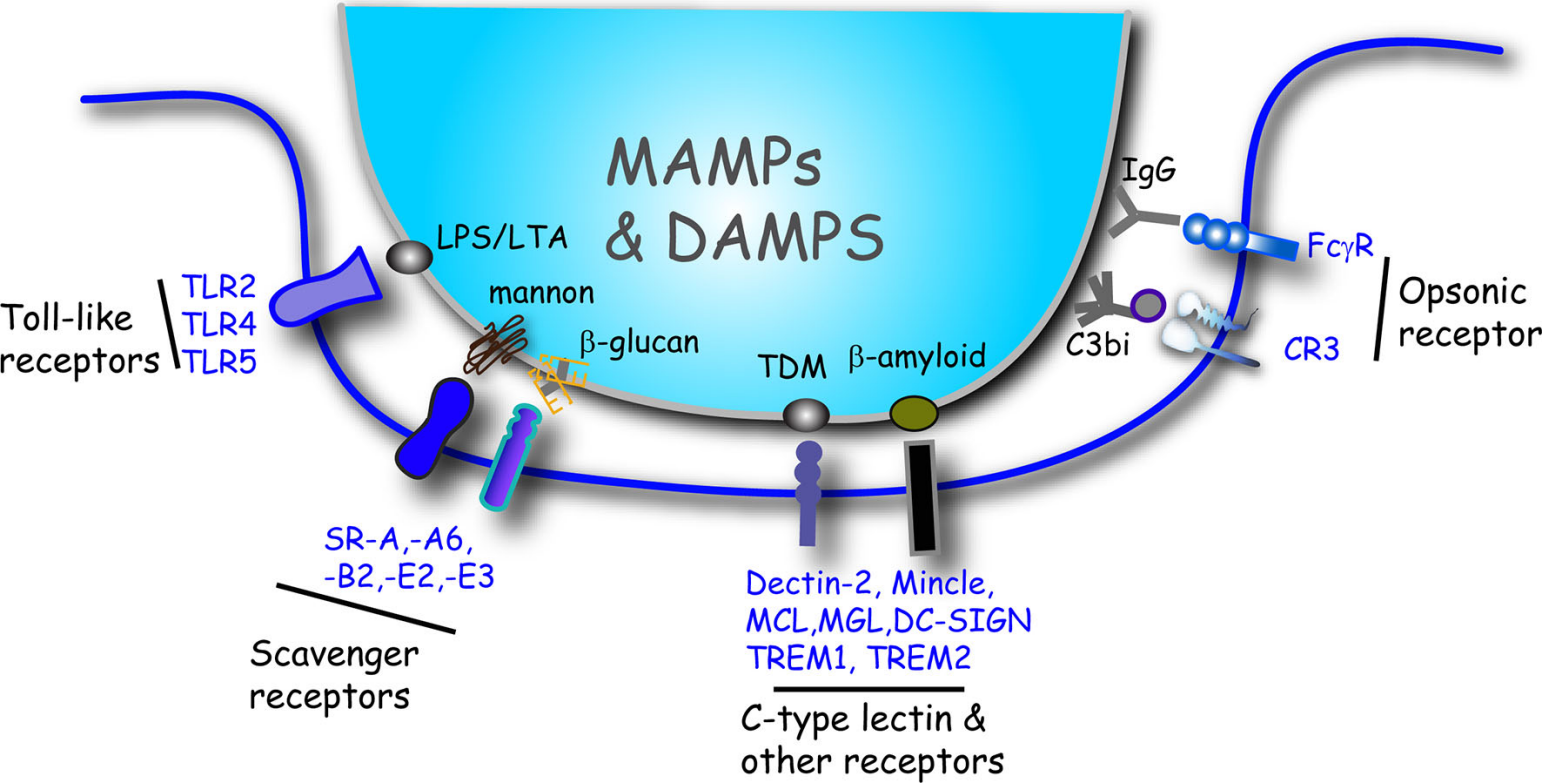
Summary illustration of the major receptor/ligand interactions that initiate phagocytosis of microbes and induce cytokine release in macrophages. Macrophages bind to targets directly or indirectly *via* opsonins. Toll-like receptors, scavenger receptors and other phagocytic receptors interact with ligands inherent to the surface of microbes that includes MAMPs and DAMPs. Some representative ligand examples are shown. Target particles that are coated (opsonized) with either IgG or C3bi bind to the Fc*γ*R or CR3 receptors, respectively. DAMP, damage-associated molecular pattern; LPS, lipopolysaccharide; LTA, lipoteichoic acid; MAMP, microbe-associated molecular pattern; SR, scavenger receptor; TDM, trehalose 6,6-dimycolate.

*Phagocytic targets*: Within the TLR superfamily, TLR2, TLR4 and TLR5 are considered phagocytic receptors in macrophages (1) (**Figure 2**). While, TLR2 often forms heterodimers with TLR1 and TLR6, TLR2 ligation is most essential for uptake of *Leishmania donovani (L. donovani)* promastigotes and *Escherichia coli* (*E*. *coli*) in macrophages (2, 3). TLR2 heterodimers recognize lipopeptides, peptidoglycan and lipoteichoic acid (LTA), lipoarabinomannan, zymosan and the hemagglutinin protein on the surface of bacteria, viruses, mycobacteria and parasites (4, 5). Alternatively, TLR4 binds to lipopolysaccharide (LPS) on the outer membrane of gram-negative bacteria to promote their uptake into macrophages (6, 7). While this review will focus on particulate microbial antigens, TLR2 and TLR4 are also the major receptors that recognize danger-associated molecular patterns (DAMPs) that are expressed on apoptotic or necrotic cells. The role of other TLRs in phagocytosis is more ambiguous, however it was recently shown that TLR5 ligation to bacteria flagella is responsible for uptake of *Pseudomonas aeruginosa* (*P. aeruginosa*) by alveolar macrophages (8). CD14 is a TLR4 co-receptor expressed on the cell surface of monocytes, macrophages and dendritic cells and is responsible for the uptake and clearance of gram-negative bacteria including nontypeable *Haemophilus influenzae* (NTHi), *Acinetobacter baumannii (A. baumannii)* and *E. coli* (9–11).

*Cytokine Induction*: Cytokine induction by TLR2, TLR4 and CD14 signaling in macrophages is strongly pro-inflammatory. Pro-inflammatory cytokine induction by soluble ligands to TLR2 and TLR4 have been well-studied and is reviewed elsewhere (4, 5, 7, 12, 13). Insight into TLR signaling events during phagocytosis of intact microbes has been gained from the use of receptor knockout mice or antibody-blocking experiments (see **Table 1** for complete list). For example, ingestion of NTHi by alveolar macrophages, null for either TLR4 or CD14, caused a marked reduction in secreted TNFα protein levels, compared to control macrophages (10). *Staphylococcus aureu*s (*S. aureus*) or *S. epidermidis* exposure to macrophages from TLR2 knockout mice resulted in attenuated TNFα and IL6 secretion, compared to control macrophages (14, 15). Additionally, bone marrow-derived macrophages (BMDMs) from TLR2 and TLR4 double knockout mice had blunted TNFα and IL6 release during uptake of the oral bacterium *Fusobacterium nucleatum* or *Aggregatibacter actinomycetemcomitans* (19). Finally, alveolar macrophages from TLR5 knockout mice had dampened IL1β production during ingestion of *P. aeruginosa* (8). Thus, similar to the extensive research on soluble ligands, stimulation of TLR2, TLR4, TLR5 and CD14 by particulate antigens induces a robust pro-inflammatory response in macrophages.

**Table 1:** Research articles investigating phagocytic receptors and cytokine production when macrophages were exposed to particulate microbial antigens.

Phagocytic receptor	Phagocytic target/ligand	Macrophage cell type	Experimental model	Receptor-induced pro-inflammatory cytokines	Receptor-induced anti-inflammatory cytokines	Literature cited
TOLL-LIKE RECEPTORS (TLRs)						
TLR2	nontypeable *Haemophilus influenzae* (NTHi)	alveolar macrophages	TLR2-/- mice	↑TNFα		(10)
	*Staphylococcus epidermidis* (*S. epidermidis*)	peritoneal macrophages	TLR2-/- mice	↑TNFα ↑IL6		(14)
	*Staphylococcus aureus (S. aureus)*	peritoneal macrophages	TLR2-/- mice	↑TNFα ↑IL6		(15)
	zymosan	bone marrow-derived macrophages (BMDMs)	TLR2-/- mice	↑TNFα		(16)
	*Candida albicans* (*C. albicans*) cell wall	human monocytes	anti-TLR2 antibody	↑TNFα		(17)
TLR4	NTHi	alveolar macrophages	TLR4-/- mice	↑TNFα		(10)
	mutant *Neisseria meningitides* (*N. meningitides)*	murine BMDMs	*N. meningitides* strain lacking LPS	↑TNFα ↑IL6		(18)
+TLR2	*Fusobacterium nucleatum* and *Aggregatibacter actinomycetemcomitans*	BMDMs	TLR2 and TLR4 double -/- mice	↑TNFα ↑IL6		(19)
TLR5	*Pseudomonas aeruginosa*	alveolar macrophages	TLR5-/- mice	↑IL1β		(8)
CD14 co-receptor	*Streptococcus pneumococci* (*S. pneumococci*), or purified *S. pneumococci* cell wall	THP1 human macrophages	anti-CD14 antibody	↑TNFα ↑IL6 ↑IL1		(20)
	group B *streptococci* type III (GBS)	human monocytes	anti-CD14 antibody	↑TNFα		(21)
	NTHi	alveolar macrophages	CD14-/- mice	↑TNFα		(10)
SCAVENGER RECEPTORS (SRs)						
SR-A	*N. meningitides*	BMDMs	SR-A-/- mice	–TNFα –IL12	–IL10	(18)
+TLR4	*N. meningitides*	BMDMs	TLR4-/- mice	↑TNFα ↑IL12		
	Trehalose 6,6′-dimycolate (TDM)-coated beads	resident peritoneal macrophages	SR-A-/- mice	↑TNFα		(22)
+TLR2/4,CD14, MARCO	TDM-beads	HEK293 cells	Co-expression of MARCO, CD14 and TLR2 or SR-A, CD14, TLR2, MD2 and TLR4	↑NF-κB activation		
SR-A6	TDM-beads	RAW264.7 cells	MARCO overexpression	↑TNFα		(22)
Macrophage receptor (MARCO)		peritoneal macrophages	MARCO-/- mice	↑IL6 ↑TNFα ↑IL1β		
+TLR2/4,CD14, SR-A		HEK293 cells	Co-expression of MARCO, CD14 and TLR2 or SR-A, CD14, TLR2, MD2 and TLR4	↑NF-κB activation		
SR-B2 (CD36)	*Plasmodium falciparum* (*P. falciparum*) malaria-parasitized erythrocytes	human peripheral blood mononuclear cells and murine peritoneal macrophages	anti-CD36 antibody	–TNFα		(23)
+TLR2	*Cryptococcus neoformans*	RAW264.7 macrophages	CD36 knockdown	↑IL1β		(24)
	*S. aureus* or *E. coli*	BMDMs	CD36-/- rat	↑IL6 ↑TNFα		(25)
	β-amyloid	peritoneal macrophages	CD36 downstream signaling kinase knockdown	↑MCP1		(26)
SR-E2 (Dectin-1)	zymosan, live *C. albicans* and *S. cerevisiae*	RAW264.7 cells	Dectin-1 overexpression	↑TNFα		(16)
	zymosan	RAW264.7 cells	Dectin-1 overexpression	↑TNFα		(27)
	*Candida glabrata* (*C. glabrata*)	thioglycollate-elicited macrophages	Dectin-1-/- mice	↑TNFα ↑IL6	↑IL10	(28)
+TLR4	*Exserohilum rostratum* (*E. rostratum*) *E. rostratum* + LPS	BMDMs BMDMs	Dectin-1-/- mice Dectin-1-/- mice	↑TNFα ↑IL1β compared to LPS		(29)
	*Aspergillus fumigatus* germ tubes	murine peritoneal macrophages	anti-Dectin-1 antibody	↑TNFα		(30)
	zymosan and soluble and particulate β-glucan	resident peritoneal macrophages, alveolar macrophages RAW264.7 cells	Dectin-1 overexpression	↑TNFα ↑TNFα		(31)
+TLR2	zymosan	HEK293 cells RAW264.7 cells with endogenous TLR2	Dectin-1 and TLR2 overexpression Dectin-1 overexpression	↑NF-κB activation ↑TNFα		(27)
SR-E3 (Mannose Receptor (MR))	*C. albicans*	thioglycollate-elicited peritoneal macrophages	MR knockdown	↑IL1β mRNA ↑IL6 mRNA		(32)
	*C. albicans*	thioglycollate-elicited peritoneal macrophages	MR-/-mice	↑TNFα ↑MCP1		(33)
	zymosan	thioglycollate-elicited peritoneal macrophages	MR-/-mice	–MCP1 –TNFα		
	*Pneumocystis carinii (P. carinii)*	human alveolar macrophages	MR-blocking ligand and MR knockdown	↑NF-κB nuclear translocation		(34)
	*P. carinii*	human alveolar macrophages	MR-blocking ligand and MR knockdown	↑NF-κB activation –IL1β –IL6 –TNFα		(35)
C-TYPE LECTIN RECEPTORS						
Dectin-2	*C. glabrata* *C. albicans* yeast *C. albicans* hyphae	peritoneal macrophages peritoneal macrophages peritoneal macrophages	Dectin-2-/- mice Dectin-2-/- mice Dectin-2-/- mice	↓TNFα ↓IL6 ↓IL1β ↓IL1β		(36)
	*C. glabrata*	thioglycollate-elicited macrophage	Dectin-2-/- mice	↑TNFα ↑IL6	↓IL10	(28)
Mincle	*Tannerella forsythia*	THP1 cells	Mincle knockdown	↑TNFα	↑IL10	(37)
	*Malassezia*	BMDMs	Mincle-/- mice	↑TNFα ↑IL6	↑IL10	(38)
	*C. albicans*	BMDMs	Mincle-/- mice	↑TNFα		(39)
+TLR2	TDM-coated beads + Pam3CSK4	BMDMs	Mincle-/- mice	↑TNFα	↑ IL10	(40)
	*Mycobacterium bovis* Bacillus Calmette–Guérin (*M. bovis* BCG)	BMDMs	Mincle-/- mice		↑ IL10	
	*M. tuberculosis* H37Rv	BMDMs	Mincle-/- mice	↑TNFα mRNA		(41)
Macrophage C-type lectin (MCL)	*M. tuberculosis*	BMDMs	MCL-/- mice	↑TNFα mRNA		(42)
OTHER RECEPTORS						
Macrophage galactose-type lectin (MGL)	*Trypanosoma cruzi*	peritoneal macrophages	MGL-/- mice	↑TNFα	↑IL10	(43)
Dendritic Cell-Specific Intercellular adhesion molecule-3-Grabbing Non-integrin (DC-SIGN)	*M. tuberculosis*	human MDMs	DC-SIGN knockdown	↓TNFα mRNA ↓IL6 mRNA ↓IL1β mRNA ↓TNFα ↓IL6		(44)
Triggering receptor expressed on myeloid cells 1 (TREM1)	*M. tuberculosis* cell lysates	BMDMs	TREM1-/- mice	↑TNFα ↑IL1β (mRNA and protein)	↑IL10 mRNA	(45)
	heat-killed *S. pneumoniae*	BMDMs and alveolar macrophages	TREM1/3-/- mice	↑TNFα		(46)
TREM2	*M. bovis* BCG	peritoneal macrophages	TREM2-/-mice	↓MCP1		(47)
	*E. coli*	peritoneal macrophages	TREM2-/- mice and TREM2 overexpression	↓IL6		(48)
OPSONIC RECEPTORS						
FcγR	IgG-coated tissue culture plates	human monocytes		↑H_2_O_2_ release		(49)
	IgG-coated beads	murine peritoneal macrophages		↑arachidonic acid		(50)
	IgG-opsonized sheep red blood cells (sRBCs)	BMDMs		–TNFα –IL1β –IL6		(51)
+TLR4	IgG-opsonized sRBCs	LPS-stimulated BMDMs		Low IgG: ↑IL12 High IgG: ↓IL12	Low IgG: ↓IL10 High IgG: ↑IL10	(52, 53)
+CD36	*P. falciparum*-infected erythrocytes opsonized with pooled patient immune serum	IFN-γ-primed human MDMs		↑TNFα ↑IL1β ↑IL6		(54)
+TLR2	heat aggregated gamma-globulins +P3C (soluble TLR2 ligand)	IFN-γ-primed human MDMs			↑IL10	(55)
Complement receptor3 (CR3)	IgM-C3bi-coated beads	murine peritoneal macrophages		–arachidonic acid		(50)
	C3b- or C3bi- coated tissue culture plates	human monocytes		–H_2_O_2_ release		(49)
	C3bi-opsonized sRBCs	BMDMs		↑TNFα ↑IL1β ↑IL6		(51)
+Dectin-1	heat-killed *Histoplasma capsulatum (H. capsulatum)*	peritoneal macrophages	CR3 and Dectin-1 single and double -/- mice	↑TNFα ↑IL6		(56)
+Dectin-1	heat-killed *H. capsulatum*	peritoneal macrophages	anti-CR3 and anti-Dectin-1 antibodies	↑TNFα ↑IL6		(57)

Phagocytic receptor and target particles are listed as well as the type of primary macrophage or macrophage cell line utilized. Experimental strategies to test receptor involvement are also briefly described. Finally, the effect of receptor stimulation on either pro- or anti-inflammatory cytokine production is summarized.

Ligation of TLR2 and TLR4 also modulates cytokine expression when other phagocytic receptors on macrophages are co-engaged by the same microbial target. This includes scavenger receptors like Dectin-1 as well as the opsonic receptors (**Table 1**) and we discuss receptor signaling cross-talk in subsequent sections.

### Scavenger Receptors (SRs)

*Phagocytic targets*: Scavenger receptors are another prominent family of phagocytosis receptors utilized by macrophages to bind and destroy microbes. These receptors include: Scavenger receptor-A (SR-A), SR-A6 (macrophage receptor with collagenous structure; MARCO), SR-B2 (CD36), SR-E2 (Dectin-1) and SR-E3 (Mannose Receptor; MR) (**Figure 2**). Scavenger receptors are PRRs that bind to several microbe-associated molecular patterns (MAMPs) and DAMPs on microbes. SR-A protects the host against the invasion of various pathogens including gram-positive bacteria such as *S. aureus*, *Listeria monocytogenes* (*L. monocytogenes*), *Streptococcus pneumoniae* (*S. pneumoniae*) and gram-negative bacteria including *Neisseria meningitides* (*N. meningitides*) (58). This protective effect is due to SR-A-mediated bacterial clearance through phagocytosis by macrophages. The scavenger receptor MARCO is expressed on macrophages of the splenic marginal zone, medullary cords of the lymph nodes and alveolar macrophages of the lung (59–62) and is used to bind *E. coli* and *S. aureus* for clearance in the spleen (63).

CD36 belongs to the class B scavenger receptor family (64) and the phagocytic targets of CD36 include *S. aureus*, *E. coli*, as well as apoptotic cells and fibrillar β-amyloid (25, 65, 66). Dectin-1 directly mediates the phagocytosis of various kinds of fungi including fungal pathogens as well as fungal particles like zymosan (67–69). With assistance from TLR2, Dectin-1 also induces uptake of *Aspergillus fumigatus* conidia by murine macrophages (70). Mannose receptors recognize and phagocytose microbial targets expressing terminal mannose residues (71–76). While some studies indicate that the mannose receptor does not discriminate between pathogenic and non-pathogenic mycobacteria (77), other evidence indicates that the mannose receptor binds and internalizes virulent *M. tuberculosis* strains more efficiently compared to attenuated bacteria strains (78, 79).

*Cytokine Induction*: Phagocytosis induced by SRs in macrophages frequently induces pro-inflammatory cytokine production and often with TLR help (see **Table 1** for complete list). While phagocytosis of *N. meningitides* is reduced in macrophages from SR-A-/- mice, the pro-inflammatory response is not impacted and is dependent on TLR4 expression and ligation to LPS (18) (**Table 1**). This is likely attributed to the inherent structure of SR-A, which does not have an intracellular signaling domain. However, peritoneal macrophages from SR-A-/- mice showed decreased TNFα production during uptake of latex beads coated with trehalose 6,6′-dimycolate (TDM), the cell wall glycolipid of *M. tuberculosis* (22), indicating a more direct role for SR-A in cytokine signaling, or the involvement of another as of yet identified signaling co-receptor. Likewise, MARCO is implicated in tethering and phagocytosis of *M. tuberculosis* but does not have an intracellular signaling moiety and requires TLR2 stimulation and signaling for cytokine induction in macrophages (22) (**Table 1**).

In macrophages, CD36 can either independently, or in cooperation with TLR2 receptors, mediate pro-inflammatory cytokine release during phagocytosis. CD36 presents bacterial LTA as well as diacylated lipoproteins to TLR2/6 heterodimers (80). Phagocytosis studies in macrophages have confirmed that CD36 signaling mediates internalization of microbes but requires TLR2 signaling for pro-inflammatory cytokine secretion (23, 24) (**Table 1**). Dectin-1 also synergizes with TLR2 for the production of pro-inflammatory cytokines in macrophages (16, 27) (**Table 1**). Phagocytosis of zymosan by macrophages occurred through either Dectin-1 or the mannose receptor, but only Dectin-1-mediated phagocytosis of zymosan led to the production of superoxide (77). As discussed later, Dectin-1-mediated cytokine induction during phagocytosis also depends on ligand density and the activation status of macrophages.

Stimulation of the mannose receptor during phagocytosis has varying effects on cytokine production in murine macrophages (**Table 1**). *C. albicans* binding to mannose receptors on thioglycollate-elicited peritoneal macrophages induced transcription of IL6 and IL1β (32), indicating a pro-inflammatory response. The mannose receptor is the main receptor for phagocytosis of unopsonized *Pneumocystis*, a pathogen that causes pneumonia (72, 81). While mannose receptor ligation to *Pneumocystis carinii (P. carinii)* induced NF-κB nuclear translocation in human alveolar macrophages, the expression and secretion of IL1β, IL6 and TNFα did not occur (34, 35). The observed effects on NF-κB translocation may be related to the different bacteria MOIs used in these studies (34, 35). NF-κB activation requires a particular threshold of signal density from PRRs, including input from MAPKs, for transcription of cytokine genes to occur (82). Other receptor involvement may also be required for pro-inflammatory cytokine production during *P. carinii* uptake, for instance through bacteria opsonization. In support of this, immune serum opsonization of *Pneumocystis* bacteria promoted significant TNFα production, compared to unopsonized bacteria, in macrophages (83).

### C-Type Lectin Receptors and Other PRR Phagocytic Receptors

*Phagocytic targets*: Dectin-2 belongs to the C-type lectin phagocytic receptors which also include macrophage inducible C-type lectin (Mincle) and macrophage C-type lectin (MCL) (**Figure 2**). Dectin-2 mediates the phagocytosis and killing of *Candida glabrata* (*C. glabrata*) by macrophages (36). Both Mincle and MCL are TDM receptors, where Mincle recognizes the carbohydrate part of TDM and MCL recognizes the lipid portion of TDM (84). Mincle ligation drives the phagocytosis of *Klebsiella pneumonia* (85), but not *C. albicans*, although interestingly Mincle is localized to both the phagocytic cup and *C. albicans*-containing phagosomes (39, 86). Mincle can form heterodimers with MCL and serves as a bridge between MCL and FcRγ. The Mincle-MCL-FcRγ complex has a much higher phagocytosis capacity for anti-Mincle- or anti-MCL-coated beads in macrophages (87).

Other phagocytic PRR receptors include macrophage galactose-type lectin (MGL) which recognizes Gal/GalNAc residues present in N- and O-glycans on glycoproteins and/or glycosphingolipids and thus MGL binds tumor antigens, apoptotic cells and foreign glycoproteins on helminth parasites (88). Dendritic cell-specific ICAM-3 grabbing non-integrin (DC-SIGN) is another C-type lectin receptor which has a carbohydrate recognition domain that recognizes glycoproteins with mannose-containing structures (89). Triggering receptor expressed on myeloid cells 1 (TREM1) and TREM2 are expressed in macrophages and other cells of the myeloid lineage (90). TREM1 mediates the phagocytosis of β-amyloid peptides by human monocytes and promotes the uptake of heat-killed *S. pneumoniae* by BMDMs and alveolar macrophages (46). TREM2, is required for the phagocytosis of *K. pneumoniae* (91), and is also responsible for the uptake of *E. coli*, *S. aureus*, zymosan and β-amyloid (Aβ 1-42) (48, 92, 93).

*Cytokine Induction*: There are conflicting studies on the role of Dectin-2 in cytokine release during phagocytosis in macrophages. In one study, Dectin-2-deficient peritoneal macrophages released more pro-inflammatory cytokines after *C. glabrata* and *C. albicans* stimulation, compared to control macrophages (36). However, another study examining thioglycollate-elicited peritoneal macrophages from Dectin-2-/- mice, showed reduced TNFα and IL6 levels and enhanced IL10 levels after *C. glabrata* infection, compared to control macrophages (28). The activation status of the macrophages may be impacting the signaling capacity for Dectin-2, which lacks signaling domains and must associate with the adaptor FcRγ chain that contains an immunoreceptor tyrosine-based activation motif (ITAM) (94).

Mincle activation can promote the release of both pro- and anti-inflammatory cytokines when macrophages engage with gram-negative bacterium, mycobacteria, fungi and inorganic particles. For instance, while Mincle was not required for the phagocytosis of the gram-negative *Tannerella forsythia* (*T. forsythia*) bacteria, Mincle ligation to *T. forsythia* promoted pro-inflammatory cytokine TNFα and anti-inflammatory cytokine IL10 release in macrophages (37) (**Table 1**). Mincle also mediated the release of TNFα, IL6 and IL10 in BMDMs ingesting the fungi *Malassezia* (38). Mincle activity can synergize with TLR2 in macrophages to promote pro- and anti-inflammatory cytokine release. As an example, BMDMs stimulated with the TLR2 agonist Pam3CSK4 produced more pro-inflammatory TNFα and anti-inflammatory cytokine IL10 when co-stimulated with TDM-coated beads, that was depended on Mincle signaling (40). While MCL has not received as much attention as other C-type lectin receptors, BMDMs from MCL-/- mice had reduced levels of TNF mRNA after *M. tuberculosis* H37Rv stimulation, compared to control macrophages (42) (**Table 1**), indicating that this receptor may signal for cytokine gene expression in a similar manner to Mincle. Similarly, little has been reported about cytokine induction during phagocytosis induced by MGL ligation, however one study of peritoneal macrophages from MGL-/- mice showed reduced TNFα and IL10 secretion during phagocytosis of *Trypanosoma cruzi* parasites, compared to control macrophages (43) (**Table 1**).

In contrast to other C-type lectin receptors, DC-SIGN signaling during phagocytosis dampens pro-inflammatory signaling in macrophages. For instance, when DC-SIGN receptor levels were experimentally downregulated in human monocyte-derived macrophages (MDMs) exposed to *M. tuberculosis*, the mRNA and secreted protein levels of TNFα, IL6, and IL1β were increased, compared to control macrophages (44) (**Table 1**). DC-SIGN is more abundant in dendritic cells, where inhibitory PRRs are more common (95), and act to the advantage of the pathogen to actively suppress host immune defenses. It is quite probable that most macrophage PRRs are strongly pro-inflammatory to reflect the front-line response of macrophages in infections.

Finally, TREM1 ligation induces a pro-inflammatory response during bacterial uptake and accordingly, TREM1 inhibition is protective in septic shock animal models (90). Macrophages from TREM1 knockout mice have attenuated TNFα and IL1β mRNA and protein levels after *M. tuberculosis* exposure (45) (**Table 1**). TNFα secretion is also reduced from TREM1/3-/- BMDMs and alveolar macrophages during uptake of heat-killed *S. pneumoniae* (46). Compared to TREM1, TREM2 is more anti-inflammatory.

Knocking out the TREM2 gene in macrophages engaging *M. bovis* BCG increased the BCG-induced release of the pro-inflammatory cytokine, MCP1 (47). Similarly, TREM2-/- peritoneal macrophages had higher IL6 secretion after engagement with *E. coli* while RAW264.7 cells overexpressing TREM2 had attenuated IL6 release after *E. coli* stimulation, compared to control cells (48). Why might there be such a variation in cytokine induction by TREM1 and TREM2? One possibility is that TREM2 does not induce complete phosphorylation of the required adaptor, DAP12. The ITAM of DAP12 becomes only partially phosphorylated if the associated receptor has a low affinity or avidity to ligand (96). Incomplete phosphorylation of the ITAM recruits the SH2 domain-containing protein tyrosine phosphatase SHP-1, leading to dephosphorylation of downstream targets of Syk (97).

### Opsonic Receptors

*Phagocytic targets*: Fcγ receptors (FcγR) bind to the fragment crystalline (Fc) region of IgG exposed on opsonized targets that have been recognized with the fragment antigen binding (Fab) portion of IgG (98) (**Figure 2**). Once ligated, clustered FcγRs use the ITAM either in the cytosolic domain of the receptor or in associated subunits (FcRγ or FcϵRIβ chain) to activate downstream signaling cascades. The complement system is activated either through the classic pathway, the alternative pathway or the lectin pathway, which leads to the generation of C3b that can bind to exposed hydroxyl and amino groups on the surface of pathogens (99). Bound C3b can also be cleaved into an inactivated form that is called C3bi (99). Phagocytes express CR3 receptors, which upon inside-out activation can bind to deposited C3b or C3bi on target cells (99, 100) (**Figure 2**).

*Cytokine Induction*: While the study of phagocytosis has centered on uptake by the opsonic receptors, there is relatively little work on their connectedness with cytokine induction in macrophages (**Table 1**). An early study of human monocytes plated on IgG-coated substrates showed a significantly higher production of H_2_O_2_ in these cells, compared to macrophages on a control surface (49). In contrast, H_2_O_2_ levels remained unchanged in monocytes plated on C3b- or C3bi-coated substrates (49). A follow-up study of murine peritoneal macrophages revealed that internalization of opsonized latex beads through Fcγ receptors, but not CRs, caused elevated arachidonic acid production, which is a potent inflammatory mediator of vascular permeability (50). This led to the belief that phagocytosis through Fcγ, but not CR3, was pro-inflammatory. However, our lab recently showed that the phagocytosis of C3bi-opsonized sRBCs increased the release of a host of pro-inflammatory cytokines including TNFα, IL1β and IL6, compared to control macrophages and those ingesting IgG-sRBCs (51) (**Table 1**). CR3 is an integrin, and we identified a role for the downstream signaling protein, calpain, in mediating this response (51).

Recent evidence suggests that FcγRs do not induce cytokine production directly, but can modulate cytokine release mediated by other receptors. For example, IFN-γ-primed human MDMs produced much higher pro-inflammatory cytokines (TNFα, IL1β, IL6) when ingesting *P. falciparum-*infected erythrocytes opsonized with pooled patient immune serum, compared to unopsonized targets (54) (**Table 1**). Complement receptors are also well-known to coordinate with TLRs for inflammatory responses when macrophages engage with targets coated with both respective ligands (101, 102). CR3 also synergizes with Dectin-1 in pro-inflammatory cytokine release when macrophages interact with microbes. For example, macrophages exposed to heat-killed *Histoplasma capsulatum* have significantly reduced TNFα and IL6 secretion when both CR3 and Dectin-1 signaling is blunted, compared to single receptor inhibition (56, 57). **Table 1** captures the available literature investigating microbial phagocytic receptor involvement in cytokine production in macrophages. We will next discuss putative cytokine-invoking signaling elements that are activated during phagocytosis and the contributions of particulate ligands and the internalization process in the inflammatory response.

## Shared Signaling Elements in Phagocytosis and Cytokine Production in Macrophages

**Figure 3:**
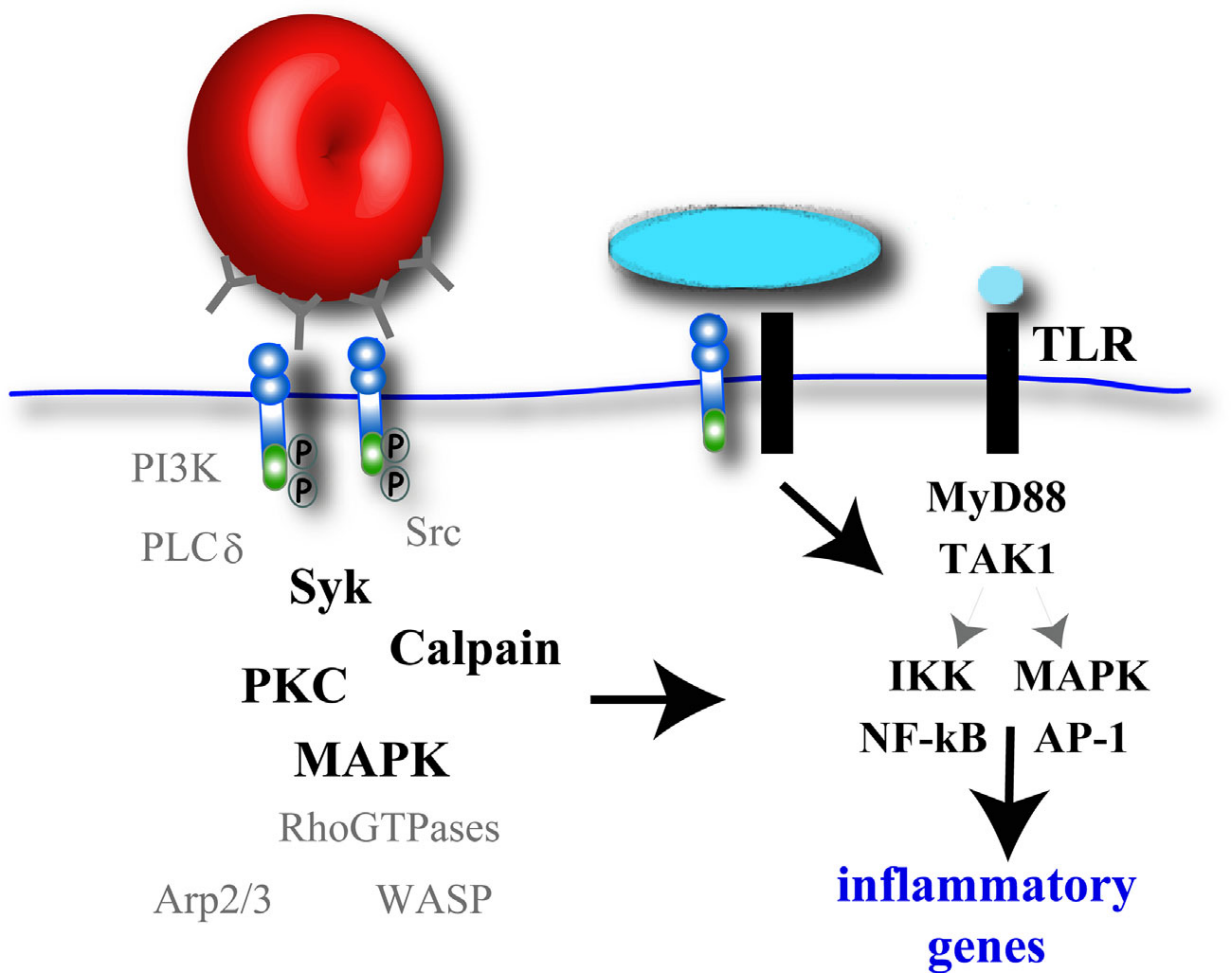
Potential signaling molecules involved in pro-inflammatory cytokine gene expression during phagocytosis. Macrophage phagocytic receptor clustering will activate F-actin-remodeling signaling elements, some kinases of which (black bold), have also been implicated in canonical TLR signaling induced by soluble ligands. While some phagocytosis receptors can independently activate an inflammatory response, other phagocytosis receptors require co-ligation of TLR receptors and subsequent TLR signaling to promote NF-κB and AP-1 activation and pro-inflammatory cytokine gene expression.

The intracellular signaling events that lead to NF-κB activation and pro-inflammatory cytokine gene expression in macrophages is best understood for TLRs and is described in several comprehensive reviews (4, 5, 103) (**Figure 3**). We have mentioned several phagocytosis receptors that cooperate with TLR-mediated signaling for cytokine induction (**Table 1**) (**Figure 3**). However, some phagocytosis receptors signal independently and induce cytokine expression in the absence of TLR ligation. These signal transduction pathways are not as well-understood due to inherent challenges when blocking receptors and the consequent impact on particle binding/internalization. Thus, it is difficult to uncouple these events and address whether the pronounced F-actin remodeling events in phagocytosis signal to the nucleus for a pro-inflammatory response. However, we are beginning to understand some of the signaling commonalities between phagocytosis and cytokine induction in macrophages. For phagocytosis, a wealth of literature exists describing the signaling events during FcγR-mediated phagocytosis (reviewed in (104, 105)). Dissecting this comprehensive signal transduction pathway allows us opportunities to identify major areas of signaling overlap (**Figure 3**) that have been substantiated with experimental evidence.

Phagocytosis is initiated by receptor engagement with the microbe and subsequent receptor clustering along the plasma membrane. The ITAMs within the receptor or adaptor proteins become phosphorylated by Src-family kinases to allow docking and activation of Syk (106, 107). Syk is also activated in the NF-κB signaling pathway induced by soluble ligands to TLR4 (108, 109) and thus represents a potential signaling hub between phagocytosis and cytokine induction in macrophages. Binding of Dectin-1 to its target leads to Syk phosphorylation which activates the caspase recruitment domain family member 9 (CARD9)/B-cell lymphoma 10 (BCL-10)/mucosa-associated lymphoid tissue lymphoma translocation protein 1 (MALT1) protein complex leading to the activation of NF-κB (107). While phagocytic receptors like FcγRIIA contain an ITAM, or hemITAM (Dectin-1), for signal transduction, others like Dectin-2 and the TREM family proteins utilize adaptor proteins containing ITAMs, which vary in their ability to induce pro- or anti-inflammatory gene expression. CD36 does not possess an ITAM and associates with the ITAM-bearing adaptor FcRγ for signaling to Syk (110). Similarly, the FcRγ ITAM becomes phosphorylated after Mincle stimulation with ligand, which in turn recruits Syk. Syk then activates the MALT1 signalosomes leading to NF-κB activation (84).

Syk activation during FcγR-mediated phagocytosis recruits PI3K and phospholipase Cγ (PLCγ) for key lipid modifications at the phagocytic cup (104, 111, 112). The production of diacylglycerol by PLCγ activity triggers calcium release that stimulates protein kinase C (PKC) and Erk1/2 and p38 MAPKs (104). PKC-ε is required for membrane delivery to the phagocytic cup (113) and also for LPS-induced 1L12 secretion (114), and thus may serve as an intersection point between phagocytosis and cytokine production in macrophages (**Figure 3**). Similarly, the MAPK family plays pivotal functions in both particle internalization and NF-κB activation. Following LPS stimulation of TLRs in macrophages, Erk1/2, p38 and JNK are activated by TGFβ-activated kinase 1 (TAK1 kinase) (115). MAPKs activate the transcription factor activator protein 1 (AP-1) leading to additional cytokine gene transcription (4, 7) (**Figure 3**). Relevant to phagocytosis, Erk1/2 is activated and required for FcγR-mediated phagocytosis in macrophages (116, 117). MARCO null macrophages exposed to TDM beads had reduced MAPK activation and TNFα, IL6, and IL1β production, compared to control macrophages (22) (**Table 1**). CD36-mediated uptake of fibrillar β-amyloid by CD36 induced Lyn signaling to Fyn and MAPK, which were necessary for pro-inflammatory reactive oxygen species (ROS) and MCP1 production in peritoneal macrophages (26). Both the amino and carboxy termini of CD36 are cytosolic and palmitoylated which targets CD36 to lipid rafts containing Src kinase family members that can activate p38 and Erk1/2 (26). Similarly, Dectin-1-mediated phagocytosis of *C. glabrata* and subsequent secretion of IL6, TNFα and IL10 in thioglycollate-elicited peritoneal macrophages was induced by the activation of MAPK and NF-κB pathways (28).

We recently identified calpain as a potential mediator of cytokine production during CR3-mediated phagocytosis in macrophages (51) (**Figure 3**). Calpain is a cysteine protease that has been shown to degrade IkBα (118), the inhibitory subunit for NF-κB (119, 120). In canonical TLR signaling, TAK1 activates the IKK complex whereby the IKKβ subunit phosphorylates IκBα. Phosphorylated IκBα is degraded by the proteasome and NF-κB is freed for translocation into the nucleus to turn on pro-inflammatory genes (**Figure 3**).

It remains to be seen whether the robust F-actin machinery involved in particle internalization (Rac1, RhoA, WAVE, Arp2/3, etc.) plays a direct signaling role in cytokine production in macrophages (**Figure 3**). F-actin depolymerizing agents have an impact on cytokine production in macrophages (described below) but these experiments do not address the role of individual F-actin signaling elements in cytokine induction and is worthy of further investigation.

## Cytokine Induction by Microbes Versus Soluble Ligands in Macrophages

Utilizing soluble ligands to study phagocytic receptor signaling allows a simplified analysis of its potential role in cytokine induction. However, soluble ligands engage fewer receptors than intact microbes and do not induce receptor clustering and cross-linking, which strongly impacts the type and amplitude of downstream signaling events. In an elegant Dectin-1 study, macrophages were exposed to either soluble β-glucans or particulate/immobilized β-glucan and assayed for pro-inflammatory responses (121). While uptake of soluble β-glucans did not induce TNFα release and reactive oxygen species (ROS) production in macrophages, both particulate β-glucan and zymosan induced a significant pro-inflammatory response. Additionally, when soluble β-glucans were adhered to 0.8 µm latex beads to promote phagocytosis, ROS production was enhanced in macrophages (121). Immunofluorescence analysis of the macrophages plated on surface-immobilized β-glucan showed that Dectin-1 adherence to large targets removed/excluded the inhibitory CD45 and CD148 proteins from the “phagocytic synapse”. Clearance of these proteins is believed to allow the Src-Syk-MAPK signaling cascade induced by Dectin-1 to induce pro-inflammatory gene expression (121). Related to this, a recent study engineered a nanoarray of TLR1/2 ligands to recreate the phagocytic synapse. The researchers were able to manipulate the spatial organization of ligands and observed that closer spacing of ligands (and presumably receptors) enhanced TNFα release in RAW264.7 cells, up to an intrinsic limit (122). While a wealth of receptor signaling information can be gleaned from studying soluble ligands, there is value in investigating large targets where the magnitude of receptor ligation and clustering may impact intracellular signaling events.

## Variations in Cytokine Signal Strength and Cross-Talk by Phagocytosis Receptors

Many signaling proteins overlap during phagocytosis and NF-κB activation pathways, yet the type of pro-inflammatory cytokines and magnitude of the secreted cytokine levels varies widely. Studies in our lab have directly compared cytokine secretion after stimulation of macrophages with different ligands. TLR signaling induced an order of magnitude higher cytokine production in macrophages, compared to CR3 or FcγR stimulation using opsonized sRBCs (51, 52). As mentioned earlier, opsonization of microbes is known to influence the cytokine production profile induced by other receptors. Antibody-opsonized targets are indicative of a measured adaptive immune response and may not signal the urgency of a new infection represented by microbes solely displaying MAMPs.

The degree of opsonization also strongly influences cytokine production in macrophages. In LPS-stimulated macrophages, increasing IgG on RBCs to saturation inhibited IL12 production and surprisingly, induced secretion of the anti-inflammatory cytokine, IL10 (53). Equivalent amounts of soluble antibody added to media had no effect suggesting that the ligand must be present at high enough densities to cluster FcγRs. This FcγR-mediated influence on TLR signaling included pronounced Erk1/2 activation, known to induce histone modifications associated with the IL10 gene promotor (123). The coinciding increase in sRBC phagocytosis at higher opsonin concentrations also begs the question of whether the enhanced internalized particles also contributed to altered cytokine gene expression. Potential receptor signaling contributions during particle uptake and from phagosome membranes are discussed in the next sections.

Dectin-1-mediated cytokine expression during phagocytosis is also dependent on the density and type of fungal ligands. Fungi with higher levels of β-glucans on the cell wall induces more Dectin-1 engagement and pro-inflammatory cytokine release, compared to fungi with lower β-glucans levels. For example, macrophage uptake of *A. fumigatus* resting conidia did not induce pro-inflammatory signals while maturing *A. fumigatus* germ tubes caused pronounced NF-κB activation and the production of TNFα and ROS in macrophages (30).

In addition to ligand density, microbes contain multiple ligands and activate PRRs and opsonic receptors of the same or different type(s). These findings underscore the importance of using intact microbes to decipher the relationship between phagocytosis and cytokine induction. As detailed in **Table 1**, complexities in cytokine induction in macrophages arise when more than one phagocytic receptor is engaged. For instance, a synergistic production of pro-inflammatory cytokine production was observed when both CR3 and Dectin-1 were engaged in macrophages ingesting *H*. *capsulatum*. The signal amplification by these receptors occurred *via* pronounced Syk activation, which then enhanced downstream Syk-JNK-AP-1 signaling (56). Dectin-1 co-ligation also magnifies TLR2 or TLR4 signaling during phagocytosis. For instance, the uptake of *Exserohilum rostratum* (*E. rostratum*) in the presence of LPS, provoked a much higher IL1β release in macrophages, compared to LPS or *E. rostratum* stimulation alone (29). Dectin-1 signaling also synergizes with TLR2 for NF-κB activation after zymosan stimulation (27). As we have discussed, MAPK may represent a common node for signaling by phagocytic receptors and parallel activation of MAPK *via* different pathways could lead to synergistic cytokine expression outcomes. The opsonization of bacteria activates both FcγR and TLR signaling pathways often leading to amplified cytokine responses in macrophages (52, 53) (**Table 1**). There likely exists a hierarchy of responses by PRRs to the threat at hand which is mediated by co-ligation of different receptor combinations.

## Role of Particle Internalization in Cytokine Induction in Macrophages

Phagocytosis is typified by dramatic F-actin remodeling and plasma membrane protrusions but is this unique morphological event required for inflammatory signaling? Some clues to help answer this question can be provided by experiments that block microbial uptake or utilize targets that cannot be internalized. Inhibition of particle internalization can be achieved with F-actin depolymerizing agents. Treatment of thioglycollate-elicited BMDMs with cytochalasin D (cyto D) to block uptake of heat-killed *S. aureus* did not impact TNF or IL10 release, suggesting that internalization was not required for TLR2/4-mediated pro-inflammatory signaling (124). In contrast, cyto D treatment to inhibit internalization of *K. pneumoniae* through TLR2 and Dectin-1 receptors, blocked secretion of the pro-inflammatory cytokine, IL8 (125). Impeding phagocytosis of gram-positive bacteria using cyto D also reduced the expectant IL12 response (126). While the latter observations were made in respiratory epithelium and monocytes, respectively, the results warrant investigation in macrophages. Cyto D is a blunt tool to block microbial internalization and we have utilized Src and Syk inhibitors in our own work to prevent particle uptake in macrophages (51). While we observed a marked reduction in pro-inflammatory cytokine secretion in drug-treated BMDMs, phagocytosis still proceeded at a lower level in Src- and Syk-inhibited cells, confounding the results (51).

Another strategy to delineate receptor signaling events is to employ “frustrated phagocytosis” assays where ligand is bound to a coverslip and thus cannot be internalized. Exploiting such an assay, robust TNFα and ROS production was seen in BMDMs engaged with plate-immobilized β-glucan, validating the cell surface as a predominant cytokine signaling locale for the Dectin-1 phagocytic receptor (121). Similarly, there was a Dectin-1-dependent secretion of TNFα when macrophages were bound to *E. rostratum*, a bacteria that cannot be internalized (29). In contrast, compelling evidence for an internalization requirement for cytokine secretion in macrophages came from a study on the rough and smooth varieties of *Mycobacteria abscessus* (*M. abscessus*), where the topology of the rough strain impedes effective engulfment by macrophages (127). Interestingly, human PMBCs engaged with rough *M. abscessus* strains produced significantly less TNF and IL10 cytokines than after engulfment of the smooth bacteria strain (127). There are a few potential reasons for this. As we discussed, shared signaling elements between phagocytosis and NF-κB activation pathways may amplify the response when microbes are internalized. Furthermore, phagocytic receptor signaling may acutely persist on the phagosome (**Figure 1**), which is the next topic of discussion.

## Phagocytic Receptor Signaling in Phagosomes

Do phagocytosis receptors signal within phagosomes for cytokine induction? As previously mentioned, it is inherently difficult to uncouple signaling events during particle uptake with cytokine gene expression, as down-regulating the receptor usually blocks internalization. However, it is well-known that after uptake of soluble ligands, internalized TLR4 can recruit TRAM and TRIF that lead to the activation of NF-κB-mediated pro-inflammatory cytokine gene expression and type I IFN gene expression (4). While TLR4 can signal at both the cell surface and within endosomes, other TLR family members signal only at the plasma membrane or from endosomal compartments (128). CARD9, an important downstream signaling element for Dectin-1, is recruited to early phagosomes where it may exert some of its pro-inflammatory effects (31). It remains to be determined if other *bona fide* phagocytic receptors mediate inflammatory signaling at the plasma membrane and/or along phagosomes (**Figure 1**). Once internalized, the newly formed phagosome organelle is quickly remodeled as it fuses sequentially with vesicles of endocytic lineage in a process known as phagosome maturation, reviewed in (105). Membrane fission events from the phagosome also occurs to collect microbe fragments for antigen presentation and to recycle receptors (129). It is unlikely that FcγR signals extensively from the phagosomal membrane as the rapid loss of F-actin from the nascent phagosomes (111) implies a termination of the signal. Interestingly, we observed recruitment and successive F-actin flashes on CR3-phagosomes that were rarely observed in FcγR-phagosomes (130). F-actin flashes also occur on some bacteria-containing phagosomes (131) and apart from mechanically fragmenting cargo (130), these F-actin accumulations may represent phagocytic receptor signaling hubs important in cytokine production.

Maturing phagosomes ultimately fuse with lysosomes for terminal cargo destruction and killing of internalized microorganisms (105). The progressive loss of receptors from the phagosome resulting from fission events likely attenuates any long-term intracellular signaling. Interestingly, mannose receptors were recruited to *C. albicans*-containing phagosomes 20 minutes after uptake and were implicated in stimulating TNFα and MCP1 production post-internalization (33). While it is not clear if phagocytic receptor signaling commonly promotes cytokine induction from the phagosome, the routing of microbial contents to lysosomes triggers additional inflammatory receptors, including intracellular PRRs (reviewed in (132)). A larger pool of ligands is obscured in intact microbes and becomes accessible only after phagolysosome formation and digestion of the microorganism. Phagosome degradation of bacteria exposes additional TLR2 ligands as well as releasing DNA to activate intracellular TLR9 and downstream inflammatory responses (133). Ultimately, phagosome membrane remodeling and saturation of intracellular receptors will cease signaling to signify removal of the microbe and defeat of the threat (134).

## Macrophage Polarization and Cytokine Induction During Phagocytosis

Macrophages exist in “resting” states and various forms of activation/polarization *in vitro* and *in vivo*. M1 macrophages are primed with microbial products like LPS and the cytokine IFN-γ and subsequently secrete many pro-inflammatory cytokines including TNFα, IL6, IL12, IL18 and IL1β (135). In contrast, IL4-induced M2 macrophages secrete anti-inflammatory cytokines such as IL8, IL10, TGFβ and MCP1 and are ascribed a wound healing role (135). While phagocytosis of pathogens in particular will polarize macrophages, it is still unclear whether pre-programmed, polarized macrophage subsets have a different inflammatory response upon encounter with a target, compared to naïve macrophages. The results surveyed in this review largely describe resting or resident macrophages, but some studies utilized PMA-treated or thioglycollate-elicited macrophages (**Table 1**), which may influence the inflammatory outcome. For instance, while resting macrophages undergoing Dectin-1-mediated uptake of zymosan did not induce NF-κB activation and TNFα production, priming of the macrophages with either GM-CSF or IFN-γ led to Dectin-1-CARD9-mediated TNFα production (31). Macrophage priming also enhanced the surface expression of Dectin-1 (31), which may account for enhanced pro-inflammatory signaling. Interestingly, we have shown that classical activation (IFN-γ + LPS) of macrophages promotes TLR4 delivery to the cell surface (136). Circulating IFN-γ is indicative of an ongoing infection, and consequent changes in phagocytic receptor display may create macrophage populations prepared for this circumstance. Our lab has also shown that classically activated macrophages have a dramatically enhanced stable microtubule population (137, 138). In M1 macrophages, these stabilized microtubule subsets have a prominent role in transporting vesicles containing matrix metalloproteinase-9, important for invasion (139). It will be of interest to determine whether phagocytosis receptor- and cytokine-containing vesicles also mobilize these stabilized tracks and whether this augments a pro-inflammatory response during phagocytosis.

## Conclusions

In this review, we summarize the numerous macrophage receptors that serve as PRRs to recognize and engulf microbes and trigger pro- or anti-inflammatory outcomes. Many of the pro-inflammatory responses during phagocytosis are activated through TLR signaling pathways alone or with other receptors collaborating with TLRs. TLRs, in concert with other phagocytic receptors, can together enhance the macrophages’ ability to target a variety of pathogens while utilizing the robust TLR signaling pathways to induce and regulate cytokine gene expression. The purpose of this review was to highlight the importance of understanding the macrophage response to whole microbes, versus soluble MAMPs, since these are the major players in infections. Future studies investigating additional receptor cross-talk during phagocytosis will likely reveal more physiological insights into cytokine outcomes for particles with multiple target moieties for macrophage engagement. Importantly, understanding how the phagocytosis machinery responsible for the uptake of large apoptotic and necrotic cells contributes to the well-described anti-inflammatory response in macrophages will add valuable insight to the field.

## Author Contributions

YF wrote the first draft and **Table 1**. RH edited the manuscript and did the figures. All authors contributed to the article and approved the submitted version.

## Funding

This project was funded by a Natural Sciences and Engineering Research Council grant (RGPIN 2017-06087) and a grant from Canadian Institutes of Health Research grant (PJT-166084) to RH.

## Conflict of Interest

The authors declare that the research was conducted in the absence of any commercial or financial relationships that could be construed as a potential conflict of interest.
